# Preferential targeting of conserved Gag regions after vaccination with a heterologous DNA prime Modified Vaccinia Ankara boost HIV vaccine regime

**DOI:** 10.1186/1742-4690-9-S2-P330

**Published:** 2012-09-13

**Authors:** A Bauer, L Podola, A Haule, L Sudi, C Nilsson, P Mann, M Missanga, B Kaluwa, L Maboko, C Lueer, M Mwakatima, S Aboud, M Bakari, J Currier, M Robb, S Joseph, S McCormack, E Lyamuya, B Wahren, E Sandström, G Biberfeld, M Hoelscher, A Kroidl, C Geldmacher

**Affiliations:** 1 Mbeya Medical Research Programme, Mbeya, Tanzania, United Republic of; 2 Karolinska Institute and Swedish Institute for Communicable Disease, Solna, Sweden; 3 Muhimbili University of Health and Allied Sciences (MUHAS), Dar Es Salaam, Tanzania, United Republic of; 4 US Military HIV Research Program, Rockville, MD, USA; 5 MRC Clinical Trials Unit, London, UK; 6 Ludwig-Maximilians University of Munich, Munich, Germany

## Background

Genetic diversity is a major challenge in the design of vaccines against variable viruses, including HIV. Engineering vaccines to induce immune responses that preferentially target conserved antigenic regions could thus contribute to improved HIV vaccines.

## Methods

During the Tanzania Mozambique HIV Vaccine trial (TaMoVac 01) phase 2a HIV vaccine trial, HIV negative Tanzanian volunteers received 3x 0.6 or 1mg intradermal injections with a multiclade, multigene DNA vaccine that included 2 plasmids encoding for clade B Gag-p37 (p17 & p24) and a recombinant Gag-p37 (clade B p17 & clade A p24). DNA vaccine recipients were boosted with Modified Vaccinia Ankara (MVA)-CMDR expressing clade A Gag that additionally covered the Gag-p15 region. Vaccine-induced T cell responses were characterized using IFN-gamma ELISpot in 45 participants after stimulation of fresh PBMC with 9 peptide pools subdividing Gag into 9 distinct antigenic regions. Data were analyzed using the Mann-Whitney test and linear regression analysis.

## Results

Antigenic regions p17 and p24 included in the DNA prime and the MVA boost were recognized with higher median magnitude than antigenic regions within p15, which was only covered by the MVA boost (p<0.0001). Antigenic regions within p24 (clade A&B prime and clade A boost) were better recognized than those within p17 (clade B prime and clade A boost; p<0.0001). We then determined the sequence homology between the MVA and the DNA Gag vaccine immunogens for each of the peptide pools. The sequence homology ranged between 91% and 0% (for p15) of total amino acids within a given peptide pool and there was a linear correlation between the sequence homology and the response rate (p=0.04, r2=0.47).

## Conclusion

These preliminary results support the hypothesis that heterologous prime-boost vaccine regimens preferentially induce immune responses targeting regions that are conserved between the priming and boosting vaccines.

